# A novel technique for thulium laser enucleation of the prostate: anterior releasing with ThuLEP (ARTh technique)

**DOI:** 10.1007/s10103-024-04071-6

**Published:** 2024-05-08

**Authors:** Hakan Öztürk

**Affiliations:** 1 Department of Urology, Izmir University of Economics, Izmir, Turkey; 2 Izmir University of Economics Medicalpoint Hospital, Yeni Girne Boulevard 1825 St. No: 12, Karsiyaka-Izmir, 35350 Turkey

**Keywords:** Thulium laser enucleation of the prostate, Holmium laser enucleation of the prostate, Benign prostatic hyperplasia, Techicque, Transient stress incontinence

## Abstract

Thulium laser enucleation of the prostate (ThuLEP) is a highly effective approach to the treatment of benign prostatic hyperplasia. We present here a description of the “ARTh Technique” and the benefits it offers in terms of improved visualization, short operation times, and easy recognition of the dissection plane, describing specifically the anterior release (AR) technique using ThuLEP(Th). Included in this retrospective study were 32 consecutive patients operated on between January 2022-November 2022. Parameters were measured before and after the procedure: the International Prostate Symptom Score(IPSS), maximum flow rate(Qmax), post-void residual urine(PVR) prostate-specific antigen(PSA), prostate volume, operation-time, morcellation-time, catheterization-time and presence of transient urinary incontinence, and compared. The median age of patients undergoing enucleation of the prostate using the ARTh technique was 64 years (range: 44–83). The median prostate volume of the patients was 83.5 ml(50–128 ml), preoperative-IPSS was 24.8(15–33), postoperative-IPSS was 7(5–11), preoperative-Qmax was 8.1 ml/Sects. (5–11.5 ml/sec), postoperative-Qmax was 26.9 ml/Sect. (20.8–34 ml/sec), preoperative-PVR was 145 ml(75–258 ml), postoperative-PVR was 36.2 ml(0–66 ml), total operation time was 51.4 min(28–82 min), enucleation time was 36.9 min(19–51 min) and morcellation time was 15.3 min(8–27 min). The ARTh technique is a safe procedure that allows the surgeon to easily recognize and adhere to the defined dissection plane, thus decreasing operation times, significantly reducing the rate of postoperative transient urinary incontinence (TUI).

## Introduction

The European Association of Urology (EAU) guidelines recommend learning and adopting an enucleation technique for prostate tissues larger than 80 ml [8]. Enucleation techniques offer decreased morbidity and mortality, and mimic open prostatectomy in principle. The holmium laser enucleation of the prostate was described for the first time in combination with a morcellation technique by Gilling et al. in 1998 [9]. Pulsed laser energy is delivered to produce a bubble cavity that aids in mechanical dissection.

The thulium laser enucleation of the prostate was described for the first time by Herrmann et al. in 2010 [10]. Unlike in HoLEP, the laser energy is delivered in a continuous beam without interruption in the ThuLEP. The wavelength of laser energy is close to 2.0 μm, and the energy at this wavelength is strongly absorbed by intracellular water molecules, which facilitates incision and mechanical enucleation, but also vaporization [11]. Studies to improve the enucleation technique are ongoing. For instance, various HoLEP techniques have been described that offer different advantages over each other, including an en-bloc technique, trilobar enucleation technique, early apical release technique, and omega technique, although only one technique has been described in the literature for ThuLEP, which is referred to as the ARTh Technique.

## Materials and methods

### Patient characteristics

Included in the study were 32 consecutive adult patients who underwent ThuLEP surgery by the same physician in a center treating patients with large prostate volumes between January-November 2022, and who met the study criteria. Patients with a history of surgery for BPH by any method, those with prostate cancer or concomitant bladder cancer, and those with neurogenic bladder or urethral stricture were excluded from the study. The patients’ age, prostate-specific antigen (PSA) level and prostate volume, International Prostate Symptom Score (IPSS) before and (1 month) after surgery, maximum flow rate (Qmax) before and (1 month) after surgery, and post-void residual urine (PVR) before and ( 1 month) after surgery were recorded. The enucleation time (min), morcellation time (min), total operation time (min), length of hospital stay, time to catheter removal, and the presence of transient urinary incontinence (at 1 month) were recorded (Table 1).

**Table 1:** Patient descriptions

Variables	Median (Min-Max) IQR	Mean ± SD
Age (years)	64 (44–83) 15	65.0 ± 10.6
PSA level (ng/ml)	2.20 (0.90–3.40) 1.1	2.2 ± 0.7
Prostate size (ml)	83.50 (50–128) 32	83.5 ± 21.7
Enucleation time (min)	36.09 (19.00–51.00) 15	36.1 ± 9.3
Morselation time (min)	15.37 (08.00–27.00) 6	15.4 ± 4.1
Total operation time (min)	51.46 (28.00–82.00) 15	51.5 ± 12.2
Cathater removal time (day)	2.00 (1.00–2.00) 0	1.9 ± 0.3
Hospitalization time (day)	2.00 (1.00–2.00) 0	1.9 ± 0.3

Data was expressed as median and inter quartile range for non-parametric variables and mean ± sd for parametric variables

**Fig. 1:**
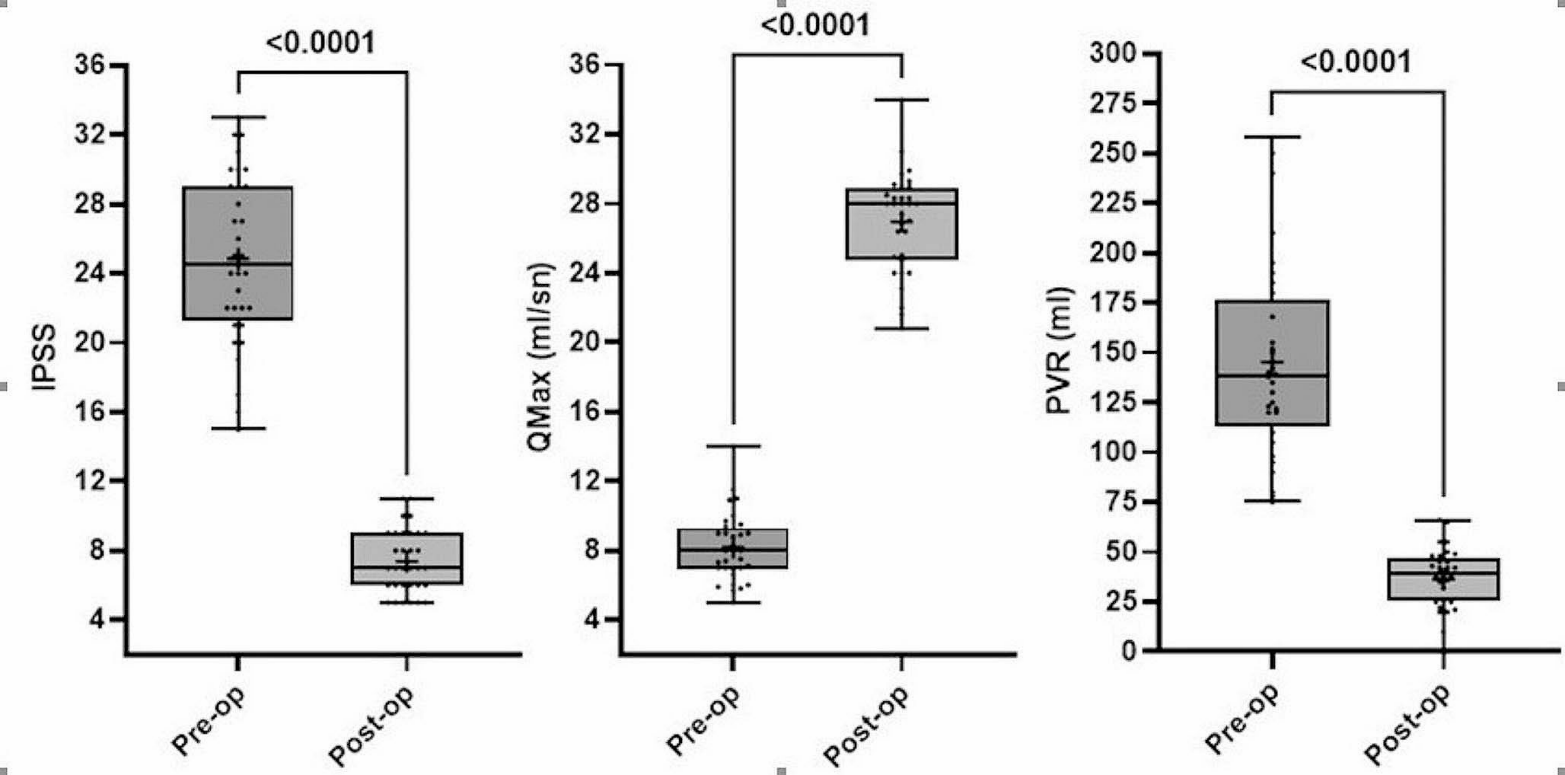
Comparison of IPSS, flow rate (Qmax), and PVR among pre and post operation

The patients’ International Prostate Symptom Score (IPSS) before and (1 month) after surgery, maximum flow rate (Qmax) before and (1 month) after surgery, and post-void residual urine (PVR) before and ( 1 month) after surgery were recorded and comparison these parameters among pre and after post operation (Table 2. ; Fig. 1.).

**Table 2:** Comparison of IPSS, flow rate (Qmax) and PVR among pre and post operation

Variables	Pre-Op (*n* = 32)	Post-Op (*n* = 32)	t	*p* Value	Normality of Residuals (*p* value)				Power	Effect Size
	Mean ± SD				Anderson-Darling	D’Agostino-Pearson omnibus	Shapiro-Wilk	Kolmogorov-Smirnov		
IPSS	24.8 ± 4.9	7.4 ± 1.8	19.91	***< 0.0001***	0.382	0.323	0.357	> 0.100	1.000	3.52
Qmax (ml/sn)	8.2 ± 2.0	27.0 ± 2.9	31.31	***< 0.0001***	0.193	0.140	0.164	> 0.100	1.000	5.54
PVR (ml)	145.1 ± 48.3	36.3 ± 16.1	14.44	***< 0.0001***	0.306	0.334	0.410	> 0.100	1.000	2.55

Paired t test used and *p* < 0.05 considered significant. IPSS; International Prostate Symptom Score, PVR; post-void residual urine

Normality of residuals p value > 0.05 shows normal distrubition. According to Cohen [1], power greater than 0.8 is appropriate

### Description of the technique

This technique was devised, described, and employed by the author, with inspiration from the open prostatectomy approach. The name “ARTh Technique” was combined by the author based on a combination of the abbreviations for anterior release (AR) and thulium (Th) laser enucleation of the prostate (ThuLEP). The surgeon has six years of HoLEP experience (70–80 cases/per year). The surgeon has four years of ThuLEP experience (80–85 cases/per year).

#### Step 1

**Fig. 2:**
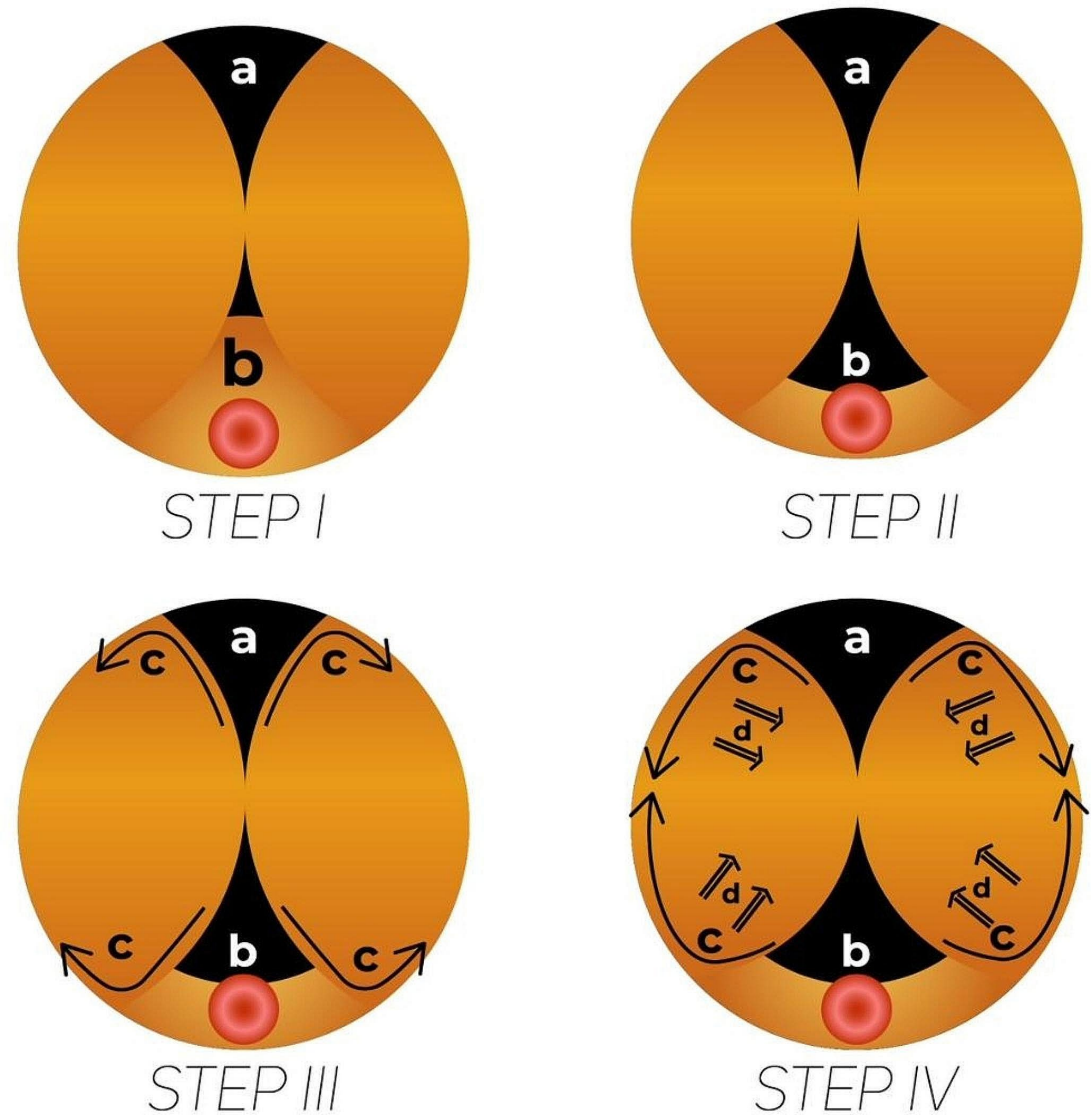
**Step I a;** AVC**-** anterior vaporization zone, **Step II b**; enucleation of the median lobe, **Step III c;** obtaining two anatomical windows and enucleation of lobes from these two different planes, **Step IV d**, hypermobility of the lobes

The most important part of the technique involves the extensive and early separation of the lateral lobes from the anterior lobe to reach the prostate capsule, as with open prostatectomy. The area measuring approximately 2–2.5 cm from an 11 o’clock to 1 o’clock position in the anterior up to the prostate capsule is called the “anterior vaporization zone (AVZ)” and vaporization is continued to the level of the verumontanum at the 12 o’clock position. This allows adaptation to the surgical anatomy by permitting the visualization of the inside of the bladder while inspecting at the level of the verumontanum (Fig. 2). This separation procedure relies on the vaporization capacity of the thulium laser. The tissues from the anterior to the prostate capsule are vaporized, and if the tissue is thick, vaporization should be continued patiently up to the prostate capsule. This can only be accomplished using a laser with vaporization capabilities. The technique is not pertinent to HoLEP, in which only a blunt dissection is performed.

#### Step 2

The dissection and enucleation of the median lobe is initiated based on the trilobar enucleation principle. The median lobe is enucleated entirely at the level of the verumontanum, and significant space is created in the prostatic urethra at the 5–7 o’clock position. Through vaporization performed at the 11–1 o’clock position, the bladder becomes visible through two different windows, at the 12 and 6 o’clock positions allowing inspection at the level of the verumontanum.

#### Step 3

Together with the enucleation of the median lobe, both lateral lobes are dissected from the prostate apex at the same surgical plane toward the 8 and 4 o’clock positions. The early release of the lateral lobes from the anterior lobe creates a bilateral anatomical space allowing mobilization to the inferior, which increases the mobility of the lobes, and lobar hypermobility is thus achieved.

#### Step 4

Sharp and clear mucosal marking and separation are achieved at the 8–11 o’clock position to the left and the 1–4 o’clock position to the right, avoiding injury to the external sphincter through manipulations during enucleation (Fig. 2).

With this technique, uniting these two surgical planes is easier than with the en-bloc technique, aided by the wider area of mobility for the right and left lobes in the prostatic urethra during enucleation. Continuation along the surgical plane is facilitated and accelerated as a result of the increased mobility of the lobe from the superior to the inferior. In addition to vaporizing the tissues, the mechanical energy produced by the thulium laser tends to follow the less resistant path, which is the plane between the adenoma and the surgical capsule. This technique allows the surgeon to approach from both the superior and inferior in cases with previous infections, and in those with dense adhesions between the adenoma and the prostate capsule resulting from previous procedures, such as prostate biopsy. Surgery can proceed through other anatomical windows in cases with adhesions that cannot be passed. In cases where these two options are not feasible, the surgeon can take advantage of the vaporization and incising capabilities of the thulium laser. When operating on patients with large prostate volumes, it is very easy to lose the surgical plane, and the return to the correct surgical plane may prolong the total operation time. Using the ARTh technique, the surgeon has the opportunity of stepping back and re-planing the surgical anatomy by inspecting at a distance if the suspicion of a deviation from the anatomical plane has been raised (Fig. 2).

An educational study conducted by Grégoire Robert et al. reported that learning this surgical technique may take up to 20 and even 50 cases, according to some publications. It has been scientifically established that 50% of new starters abandon the enucleation method for one reason or another [2–4].

The complementary equipment for this technique included a 25-degree optic and a 24-Fr continuous-wave resectoscope (Karl Storz GmbH, Tuttlingen, Germany). The outer sheath of the resectoscope was compatible with the morcellation nephroscope to be used thereafter. A Thulium: YAG laser (RevolixTM 150 W, LISA Katlenburg, Germany) was used as the source of thulium laser energy, and a 550-micron-thick fiber was used for the energy transfer (RigiFibTM, LISA, Katlenburg, Germany). The power of the Thulium: YAG laser ranged from 0 to 150 watts (W), with 80 W used for enucleation, 40 W for coagulation, and 120 W for anterior zone vaporization. The energy of 80–120 W was sufficient for the cutting function, although this varied depending on the tissue hardness. A 0.9% physiological saline solution was used for irrigation throughout the procedure. The inner sheath of the 24-Fr continuous-wave resectoscope was removed and visualization was acquired using a 24-Fr nephroscope compatible with the outer sheath during the removal of the enucleated tissues, and the morcellation procedure was completed using a Hawk Jaws tissue morcellator (Hawk, Minitech Co. China).

## Results

A total of 32 patients were analyzed retrospectively, with a median age of 64.0 years (IQR = 15 years) and mean age of 65.0 ± 10.6 years. The median prostate volume of the patients was 83.5 ml (IQR = 31 ml) and mean 83.5 ± 21.7 ng/ml; the median PSA level was 2.2 ng/ml (IQR = 1.1 ng/ml) and mean 2.2 ± 0.7 ng/ml; the mean IPSS of the patients before surgery was 24.8 ± 4.9; the median IPSS at one month was 7.4 ± 1.8. Uroflowmetry parameters were recorded preoperatively, and the mean preoperative Qmax was 8.2 ± 2.0 ml/sec; the mean Qmax at postoperative one month was 27.0 ± 2.9 ml/sec; the mean preoperative PVR measured by USG was 145.1 ± 48.3 ml; the mean postoperative PVR was 36.3 ± 16.1 ml; the mean enucleation time of the patients was 36.1 ± 9.3 min; and the mean morcellation time was 15.4 ± 4.1 min. The mean total operation time, being the total of the enucleation time and the morcellation time, was 51.5 ± 12.2 min. There was no statistically significant change in the patients’ hemoglobin values. The median length of hospital stay was 2 days, and the median time to catheter removal was 2 days. Transient stress incontinence was noted in only two patients included in the study, and 30 patients suffered no urinary incontinence within the postoperative 1 month.

### Statistical analysis

Statistical analyzes were performed with IBM® SPSS® 26 (IBM Corp. Released 2019. IBM SPSS Statistics for Windows, Version 26.0. Armonk, NY: IBM Corp) and GraphPad 8.3.0 [5]. The conformity of the variables to the normal distribution was examined using analytical methods (Kolmogorov-Smirnov/Shapiro-Wilk tests). Descriptive analyzes were given as mean ± standard deviation and median, min-max and IQR for continuous data. Descriptive statistics were made by giving frequency and percentage values ​​of categorical variables obtained from sociodemographic and clinical information. In continuous data (urological parameters etc.), paired t-test was used for independent groups when it showed normal distribution to compare binary groups (pre-op vs. post-op). Pearson’s or Fisher’s exact chi square test used for comparing categorical variables. A p-value below 0.05 were considered statistically significant.

## Discussion

Multiple enucleation methods have been described for HoLEP, including trilobar enucleation, the en-bloc technique, and early apical release techniques, each of which has its own advantages and disadvantages. The endeavors to describe new enucleation techniques are driven by the individual efforts and skills beyond standard surgical skills for the performance of this surgical procedure, and this development process indicates that standards for enucleation surgery have yet to be established. Despite the ongoing efforts to describe new techniques for HoLEP, no new technique has been described for ThuLEP. ThuLEP surgical approaches are based on HoLEP techniques, though it needs to be kept in mind that ThuLEP is a combined method, the components of which cannot be clearly distinguished. That is, the enucleation is performed using both blunt and mechanical dissections, in combination with both vaporization and incision procedures [11]. The functional status resulting from the nature of this surgery is an advantage for ThuLEP and has been described as revolutionary by Bach et al. [6], and the author of the present manuscript agrees. The present study describes a new technique that takes advantage of this unique characteristic. The most challenging aspect of enucleation surgery is the loss of the surgical plane. In the ARTh technique, the bladder is made visible through two different windows at the level of the verumontanum at the 12 and 6 o’clock positions through the enucleation of the median lobe constituting the part of the trilobar enucleation together with anterior vaporization from the 11–1 o’clock positions. These two gateways allow the surgeon to maintain a consistent surgical plane. The enucleation of the tissues at the 6 and 12 o’clock positions facilitates blunt dissection as a result of the mobilization of the lateral lobes.

In a meta-analysis by Hartung et al. involving 556 studies comparing the data on HoLEP and ThuLEP, the decrease in hemoglobin levels was considerably less significant in ThuLEP than in HoLEP [11]. Although the reduced hemorrhage with ThuLEP is remarkable in terms of the hemodynamic parameters, hemorrhage implies a prolonged coagulation time when acquiring a direct view of the surgical site. This means that the overall operation time was unfavorably affected. In fact, bloodless surgery means better surgical visibility, allowing the easier location and maintenance of the correct surgical plane. The rate of blood transfusion was 2% for HoLEP in this meta-analysis. In a comparative study, Becker et al. reported shorter operation times with ThuLEP [7]. The results of the meta-analysis by Hartung et al. indicate that even though the functional outcomes of HoLEP and ThuLEP were similar, the hemorrhage was significantly less and operation times were shorter with ThuLEP.

The median operation time was 72 min and the median prostate volume was 46.6 ml in a series of 71 patients undergoing ThuLEP in a 2012 study by Zhank et al.; 50 min and 82.5 ml in a series of 48 patients reported by Becker et al.; and 63.9 min and 90.2 ml in a series of 115 patients reported by Bozzini et al. In a more recent study of ThuLEP conducted by Zhank et al. in 2020, the median operation time was 71.4 min and the median prostate volume was 91.8 ml [7, 12–14]. Using the ARth technique, the present study reported a median operation time of 51.4 min and a median prostate volume of 83.5 ml.

Prostate volume is the parameter that mostly affects operation times. Our results are quite similar to those reported by Becker et al. but significantly shorter than those reported in other studies. Our data on operation times supports the data provided by meta-analyses in the literature. In a large series of 801 patients comparing the en-bloc and early apical release methods described for HoLEP in a 2023 study, the median operation time was 91.9 min and the median prostate volume was 103 ml in 571 patients in the en-bloc group, compared to 100.5 min and 104 ml in 230 patients in the early apical release group [15]. The study conducted by Ericson et al. reported early apical release to be associated with less hemorrhage and a shallower learning curve than the en-bloc technique [15]. This large series of patients reveals that despite the presence of new techniques described for HoLEP, the operation times remain far higher than those reported for ThuLEP. The operation times with HoLEP will continue to lag behind those reported for ThuLEP, regardless of the technical advances, as the weapons are not comparable, according to the author. The use of pulsatile burst energy balloons during the dissection, the increased bleeding during surgery and the blocked view of the surgical site due to this bleeding while trying to maintain a consistent surgical plane, and the use of bipolar cauterization equipment to achieve hemostasis in some instances are the limiting factors for HoLEP procedures.

In the data of 579 patients reported in a meta-analysis in literature, the preoperative IPSS of the patients was in the 17.9–24.6 range, while the postoperative IPSS ranged between 3 and 8 in the same patient group [11]. The median preoperative IPSS was 24.8 and postoperative IPSS was 7 in the present study, which is consistent with the values reported in the literature. In the most recent meta-analysis involving patients undergoing either ThuLEP or HoLEP, the preoperative Qmax ranged from 6.6 to 12.2 ml/sec and the postoperative Qmax ranged from 19.4 to 26.6 ml/Sect. [11]. In the present study, the median preoperative Qmax was 8.16 ml/sec and the median postoperative Qmax was 26.95 ml/sec, consistent with the literature. The residual urine volumes derived from studies comparing enucleation techniques across a large series ranged from 64.6 to 172.7 ml in the preoperative period, and from 7.5 to 42.1 ml in the postoperative period. The percentage change in these studies was between − 70% and − 80%. The median preoperative PVR, measured by USG, was 145 ml in the present study, and the median postoperative PVR was 36.25 ml. The percentage change in the amount of residual urine 1 month after surgery was − 75%, which is consistent with the data in the guidelines [8].

Transient urinary incontinence (TUI) occurred in only two patients undergoing ThuLEP using the ARTh technique, corresponding to a rate of 6.2%. According to the data presented in a meta-analysis comparing HoLEP with ThuLEP, TUI would seem to be more common in those undergoing HoLEP [11], although this data was regarded to have a low evidence level in the meta-analysis by Hartung et al., and so suggested that caution be exercised when interpreting this result. Although there are many studies in the literature comparing HoLEP with ThuLEP, only three studies have specifically reported TUI. The study by Bozzini et al. did not report whether incontinence was transient or chronic, and in the present study also it is unknown whether the urinary incontinence noted in two patients (6.2%) at 1 month was transient or chronic [13].

The authors of the above-mentioned meta-analysis regarded this condition as transient urinary incontinence. The reasons for a higher incidence of TUI in the HoLEP cohort could be related to the strictly anatomical enucleation in the HoLEP group, the more radical ablation of tissues in the prostate apex, in close proximity to the sphincter, and the deeper tissue penetration of HoLEP than with ThuLEP [16]. Although the data indicating a high incidence of TUI has a low evidence level and so needs careful evaluation, surgeons performing HoLEP in search of new techniques in this regard may be a reality that may not have been reflected to the literature, as some authors in the literature claim that modifications to HoLEP, such as apical release and mucosal sparing, decrease the incidence of TUI [17]. According to the authors of the present manuscript, the reason for the lower incidence of TUI associated with the ARTh technique could be the reduced influence on the sphincter from enucleation and manipulation due to the lobar hypermobilization achieved beforehand, the improved sparing of the mucosa due to the cutting function in ThuLEP and the short operation times.

In conclusion, the ARTh technique is a novel approach described for ThuLEP in which operation times are considerably shorter compared to the en-bloc and early apical release techniques described for HoLEP, and also significantly shorter than those reported in ThuLEP series. The functional outcomes of ARTh technique are consistent with those noted in the guidelines and in the meta-analysis data. However, urinary incontinence is considerably less common in the ARTh technique. This technique should be employed in larger series considering the ease at which it can be learned and employed.

### Limitations

The relatively small number of patients, the lack of a control group and the short follow-up period for incontinence can be regarded as the limitations of the study. The postop results may be related to both this method and the surgeon’s experience.
